# Military experience predicts military multitasking better than laboratory measures in officer cadets

**DOI:** 10.1186/s41235-025-00639-0

**Published:** 2025-06-02

**Authors:** Yannik Hilla, Maximilian Stefani, Elisabeth V. C. Friedrich, Wolfgang Mack

**Affiliations:** 1 Department of Human Sciences, Institute of Psychology, General Psychology, University of the Bundeswehr Munich, Werner-Heisenberg-Weg 39, 85577 Neubiberg, Germany; 2 Department of Psychology, University of Zurich, Zurich, Switzerland; 3 University of Sustainability Vienna–Charlotte Fresenius Privatuniversität, Vienna, Austria

**Keywords:** Military performance, Working memory, Bayesian statistics

## Abstract

Whether or not it is possible to predict military performance using laboratory measures constitutes an important question. There are indications that humans possess a common multitasking ability enabling them to perform complex behaviors irrespective of task requirements. Working memory processing abilities likely illustrate cognitive substrates thereof. Thus, it should be possible to predict military performance by means of laboratory multitasking via working memory processing abilities. To investigate this, we recruited 40 officer cadets and assessed their laboratory multitasking proficiency using the Multi-Attribute Task Battery and their performance in a simulated military operation. We then tested if the laboratory measure predicted their military performance and if this relationship was mediated by working memory processing abilities using Bayesian procedures. We also controlled if demographics, military characteristics, media preferences, or social/personality traits affected any of these measures. In contrast to our expectations, the associations between laboratory and military multitasking and working memory were weak. Furthermore, the participants did not display multitasking decrements but improvements as a function of time on task in the military setting. Moreover, we found a positive association between the time officer cadets had already served in the military and military performance. We discuss the role of learned task representations in this regard and conclude that it might be more reasonable to investigate cognitive functions as co-variates of associations between military characteristics (e.g., military service duration) and military performance in future research than to focus on laboratory measures as predictors of military performance.

## Public significance statement

Laboratory research aims to identify predictors of military performance to improve military efficiency. This illustrates a challenging endeavor given that laboratory settings struggle to replicate realistic military conditions. Some research indicates that laboratory multitasking abilities illustrate promising predictors of military performance. Allowing to temporarily store, maintain, and manipulate information, working memory constitutes an essential capacity in this regard. Thus, laboratory multitasking should predict military performance as a function of working memory capacity. We investigated this in 40 officer cadets. But neither correlated their military performance sufficiently with their laboratory multitasking performance nor either one of these measures with working memory. However, military performance was related to the time the officer cadets had already served in the military. We conclude from this that military experience (as a function of training and/or service duration) appears to affect soldiers’ military multitasking abilities so strongly that laboratory measures lose their predictive power. Therefore, it may be more reasonable to focus more on the role of military characteristics (e.g., training (progress), military service (duration), etc.) in military performance and which and how cognitive functions affect said associations in future research than on the predictive power of laboratory measures.

## Introduction

Being able to perform two or more tasks at the same time (*multitasking*) illustrates a considerable skill in everyday life. In doing so, we are able to cook, drive, and to complete complex work assignments. At the same time, it is commonly observed that performance declines in conditions of multitasking compared to when tasks are performed separately. For instance, it can be more difficult to cook while having a conversation compared to cooking in silence. Similar performance decrements have been experimentally replicated using the so called *dual-task paradigm approach* (where multitasking performance is operationalized based on performance differences between single- and multi-task conditions) and related to a capacity limitation (Pashler, 1994). The exact nature of said capacity limitation is debated but attention/cognitive control and working memory processing abilities have been discussed as essential determining factors thereof (see, Musslick & Cohen, 2021, for an overview of multitasking theories). One approach even suggests that these abilities may constitute a cognitive substrate of a common multitasking ability accounting for performance in any multitasking condition: especially, Redick et al. (2016) put this idea forward after observing that several different multitasking measures could be used to fit one common multitasking ability variable that was best explained by attention control and working memory variables.

If this was true, individual (attention) control and working memory processing abilities would play a more essential role for predicting and training multitasking abilities than accounting for task requirements. Given that the latter is challenging because task requirements are diverse and may change as a function of time and needs, while cognitive functions can be assessed relatively easy under laboratory conditions, the concept of a common multitasking ability for predicting performance is appealing. This applies especially to high-risk environments where multitasking decrements may have detrimental consequences, as in the military. There, many tasks and operations require multitasking proficiency (also referred to as *military multitasking*): for instance, pilots must monitor control panels and respond to radio signals while piloting an aircraft; command and control requires managing and executing orders in addition to conducting monitoring tasks (e.g., radar surveillance); and ground soldiers need to monitor their environment, execute orders, and be ready to act at an instant (see, Chérif et al., 2018, for a review).

There are indications that a common multitasking ability—especially as a function of working memory processing abilities—may play an essential role in military multitasking. Hambrick et al. (2011), for instance, found that laboratory multitasking performance as operationalized by the *SynWin* (formerly *SYNWORK*) task battery (Elsmore, 1994) constitutes a predictor of *armed services vocational aptitude battery* performance in Navy sailors. Furthermore, they found that this association was mediated by their working memory processing abilities (based on *n*-back task performance). Thus, they replicated their previous observation that working memory processing abilities represented a strong predictor of multitasking proficiency—even stronger than processing speed (Hambrick et al., 2010). Additionally, Colom et al. (2010) found that working memory capacity (based on span task and dot matrix performance) predicted multitasking (assessed via a funnel and a divided attention task) better than intelligence scores in applicants for an air traffic control training; and that this relation between working memory and multitasking was independent of intelligence.

Likewise, there is also research opposing this account: for instance, Hamilton et al. (2019) found that exclusively cognitive training of inhibitory control, quick decision-making, and processing speed in contrast to spatial and visual working memory training improved “shoot/don’t shoot” task performance in police officers. Similarly, response inhibition training has been shown to improve shooting simulation task performance in civilians (Biggs et al., 2015). In both cases, there was a remarkable similarity in task requirements between training regimes and test tasks highlighting the significance of task requirements for training multitasking. Besides, there are doubts as to whether laboratory working memory training improvements may even transfer to military environments, given that such improvements commonly only transfer to similar task conditions (*near transfer effect*) (Blacker et al., 2019). Thus, accounting for task requirements may be more vital for predicting military multitasking than working memory processing after all.

Hilla et al. (2024) addressed this inconsistency and investigated which one of two approaches constituted a better account of predicting military multitasking: either laboratory dual-task decrements (when math problems and radio signals had to be solved and memorized simultaneously) or low-fidelity flight simulation task performance assessed via the open-source version of the *Multi-Attribute Task Battery* (MATB; Cegarra et al., 2020). Military multitasking had been operationalized based on a simulated military operation, whereby officer cadets were instructed to solve similar math problems and to memorize similar radio signals as in the laboratory while shooting at ring targets. Thus, the laboratory paradigm shared task requirements with the military task but the MATB did not. Furthermore, Hilla et al. (2024) controlled for the officer cadets’ working memory abilities (using digit span tasks). They found that the officer cadets’ MATB performance predicted their military performance better than laboratory multitasking based on the dual-task paradigm approach despite little overlap in task characteristics. A common multitasking ability may explain this effect. However, there were only weak correlations between MATB performance and working memory. Thus, the underlying mechanism of this association between laboratory and military multitasking was unclear.

The authors discussed if digit span task performance may have been insufficient to operationalize working memory abilities due to a lack of complexity (Hilla et al., 2024). Thus, a more sophisticated measure of working memory processing may have mediated the association between MATB and military multitasking performance. But of course, it is also possible that working memory might indeed not play an essential role in the association between MATB and military multitasking. In fact, there is meta-analytic evidence questioning the significance of working memory and emphasizing the role of executive and attention (control) functions in multitasking behavior (Himi et al., 2019). Furthermore, Redick et al. (2016) showed that the relationship between working memory and multitasking performance was partially mediated by attention control. In sum, these results imply that that the role of working memory processing abilities in (military) multitasking may be overestimated.

However, this conclusion may be premature: It is true that Redick et al. (2016) found that the relationship between working memory and multitasking had been partially mediated by attention control, but they also found that the remaining part was mediated by a capacity variable based on working memory performance measures. Thus, Redick et al.’s (2016) observation may actually highlight the significance of working memory in multitasking. In this regard, it should also be noted that even though working memory set-size measures were used to fit this capacity variable, (working memory) capacity unlikely arises from a mere set-size limitation but rather from challenges of concurrently maintaining, recalling, and manipulating (task) representations of at least one task (Musslick & Cohen, 2021; Oberauer et al., 2016). Furthermore, working memory capacity unlikely constitutes just a passive storage system with attention/executive control functions managing said representations but working memory may also inherently contribute to executing these operations self-sufficiently (Barak & Tsodyks, 2014; Hilla et al., 2025; Oberauer et al., 2016). Given that it is challenging to dissociate these contributions on a conceptual level without computational modeling and/or additional neurophysiological assessments, this might explain why Himi et al. (2019) could not find an explanatory effect of working memory on multitasking behavior when controlling for executive functions, relational integration, and divided attention. In sum, working memory processing abilities might yet constitute a major constraint of a common multitasking ability and thereby of military multitasking (Colom et al., 2010; Hambrick et al., 2011; Hilla et al., 2024). However, a replication of the original effect (an association between laboratory and military multitasking) described by Hilla et al. (2024) is required to test this account.

In this regard, controlling for additional co-variates and refining the original experimental design to create more realistic military assessment conditions is necessary to prevent confounding effects. Chérif et al. (2018) pointed out that especially the role of personality and social traits should be considered more in military investigations. Klee and Renner (2016), for instance, found that German soldiers appear to exhibit lower neuroticism, openness to experiences, and agreeableness but higher extraversion and conscientiousness scores than civilian individuals. This may affect their (military) multitasking abilities, given that Himi et al. (2023) came to the conclusion that conscientiousness, openness to experiences, and *polychronicity* (i.e., the personal preference for performing several tasks at once) may have a moderating effect on the relationship between cognitive abilities and multitasking. Furthermore, Mathieu et al. (2000) found that the ability of sharing mental models, which requires empathy, perspective taking, emotional regulation, and self-monitoring, impacted on dyadic flight-combat simulation task performance. Thus, social traits should be controlled for in investigations of military performance. Besides, media usage has been observed to affect (military) multitasking: video gaming, for instance, has been frequently shown to improve cognitive functions and military multitasking, e.g., flight (simulation) performance (Blacker et al., 2019; Chiappe et al., 2013; Gopher et al., 2017; Lu et al., 2022). In contrast, media multitasking (i.e., frequently using at least two types of media simultaneously) has been shown to negatively affect working memory, attention (inhibition), and switching (Ophir et al., 2009), which is detrimental for multitasking (Musslick & Cohen, 2021). On top of that, a priori military experience probably affects how effectively participants perform military multitasking and should be controlled for: Hilla et al. (2024), for instance, found that alongside MATB performance officer cadets’ military service duration predicted their military performance.

Thus, the goal of this study was to replicate the effect according to which (1) *laboratory multitasking* performance predicts *military multitasking* (Hilla et al., 2024); and to demonstrate (2) that this relationship is mediated by *working memory processing abilities* (Colom et al., 2010; Hambrick et al., 2011; Redick et al., 2016). For this, we recruited 40 officer cadets to undergo three assessments (*online questionnaire*, *laboratory assessment*, and *military assessment*). Moreover, we controlled for personality traits (*extraversion*, *agreeableness*, *conscientiousness*, *neuroticism*, and *openness to experiences*; and *polychronicity*) (Himi et al., 2023), social traits (*empathy*, *perspective taking*, *emotion regulation*, and *self-monitoring*) (Mathieu et al., 2000), media preferences (*video gaming*, and *media multitasking*) (Blacker et al., 2019; Chiappe et al., 2013; Gopher et al., 2017; Lu et al., 2022; Ophir et al., 2009), and a priori military experience (*military service duration*) (Hilla et al., 2024).

## Methods

### Participants

Using G*Power (Faul et al., 2009), we conducted a sample size analysis to determine how many data points were required to find a moderate-to-large effect (*r* ~ 0.40) with 80% statistical power and a 5% alpha error. This analysis suggested that data of at least 34 individuals would be needed. Assumptions about effect sizes were based on previous findings (Hambrick et al., 2011; Hilla et al., 2024). This illustrated just an initial appraisal: mediation analysis commonly requires a larger sample size but we lacked vital prior information on all anticipated effect sizes to compute a sample size analysis in this regard. To compensate for this, we conducted Bayesian procedures.

We collected data of 40 officer cadets. Four individuals did not participate in the last assessment. Thus, data of 36 participants were used for further analyses. Most of the 36 participants were (under-)graduate students. They were between 20 and 30 years old (*M* = 23.19, *M*_Bayes_ = 23.42, 95% CI [22.42, 24.42], *age* ~ exponentially modified Gaussian). The majority were male (*n* = 32), right-handed (*n* = 34), and pursuit studies in Social Sciences (*n* = 21). Moreover, most of them were part of the Infantry (*n* = 18) and had served in the military for at least one year (*M* = 3.23, range [1, 12], *M*_Bayes_ = 3.03, CI [2.8, 3.25], *military service duration* ~ exponentially modified Gaussian). Additionally, 8 individuals had flight experience, whereby 4 had acquired flight licenses. Furthermore, 24 individuals were classified as video game players (Green et al., 2017). Their average play time was 11.18 h/week (in the past 12 months) (range [2, 32], *M*_Bayes_ = 9.82, CI [8.08, 11.53], *video game play time* ~ exponentially modified Gaussian). Participants did not receive a monetary compensation but could acquire student lab tokens for participation. Individuals provided written informed consent prior to participation and this study was approved by the local Ethics Committee.

## General procedure

Volunteers were recruited using the *ORSEE* software (Greiner, 2015). The *online questionnaire* was completed from home via *SoSci* survey (Leiner, 2014). The *laboratory assessment* was conducted in a university laboratory equipped with sound proof cabins. Stimuli were presented on a 27 inches EIZO® color monitor with a 144 Hz frame rate and a 2560 × 1440 pixels resolution, using *OpenSesame* (Version 3.3.14; Mathôt et al., 2012), *Presentation* ® (Neurobehavioral Systems), and in-house *Python* software. The *military assessment* was conducted in an off-campus training facility. The assessments were conducted on different days between April and May in 2023 and 2024.

## Online questionnaire

### Autism spectrum quotient

The *online questionnaire* comprised the Autism Spectrum Quotient (AQ) to assess *autistic personality traits* (Baron-Cohen et al., 2001). This was because the AQ includes—among other measures—measures of communication skills, attention to detail and attention switching abilities. Moreover, high AQ scores have been observed to go along with differences in working memory processing (Friedrich et al., 2023). Indeed, the participants displayed an elevated average AQ score of 28.49 (> 20) (*range* [13, 42], *M*_Bayes_ = 28.23, CI [26.18, 30.29], *AQ* ~ Gaussian) (Baron-Cohen et al., 2001).

### Empathy

On top of that, *empathy* was assessed using items of the *perspective taking* and *empathic concern* scales (Davis et al., 1980). We chose these scales because they have been employed in previous investigations of multitasking (Mills et al., 2015). Larger values indicate a larger propensity for empathy and perspective taking. The participants displayed an average *perspective taking* value of 3.46 (range [2, 4.86], *M*_Bayes_ = 3.47, CI [3.15, 3.78], *perspective taking* ~ Gaussian) and an average *empathic concern* value of 3.14 (range [1.29, 4.71], *M*_Bayes_ = 3.15, CI [2.84, 3.46], *empathic concern* ~ Gaussian). These were larger than the expected values ranging from 2.40 to 3.10 (average divided by number of items) (Davis et al., 1980).

### Personality traits

*Extraversion*, *agreeableness*, *conscientiousness*, *neuroticism*, and *openness to experiences* were assessed using a short version of the *Big Five Inventory* (BFI-K) (Rammstedt & John, 2005). These personality traits have been identified as potential moderators of the relationship between cognitive functions and multitasking (Himi et al., 2023). Larger values indicate a larger propensity for extraversion, agreeableness, conscientiousness, neuroticism, and openness to experiences. In civilians, the average scale scores should range between 3 and 4 (Rammstedt & John, 2005). The participants displayed average scale scores of 3.19 (range [1.5, 5], *M*_Bayes_ = 3.17, CI [2.91, 3.43], *extraversion* ~ Gaussian), 2.63 (range [1, 4], *M*_Bayes_ = 2.62, CI = [2.38, 2.87], *agreeableness* ~ Gaussian), 3.42 (range [1.25, 4.75], *M*_Bayes_ = 3.4, CI = [3.15, 3.64], *conscientiousness* ~ Gaussian), 2.54 (range [1, 5], *M*_Bayes_ = 2.54, CI = [2.29, 2.79], *neuroticism* ~ Gaussian), and 3.66 (range [2, 5], *M*_Bayes_ = 3.62, CI = [3.37, 3.87], *openness for experiences* ~ Gaussian) for *extraversion*, *agreeableness*, *conscientiousness*, *neuroticism*, and *openness for experiences*, respectively. Thus, their average scale scores fell within the established range.

### Polychronicity

*Polychronicity* was assessed using the *Inventory of Polychronic Values* (IPV) (Bluedorn et al., 1999). Also, this personality trait has been identified as potential moderator of the relationship between cognitive functions and multitasking (Himi et al., 2023). Larger values indicate a larger affinity for *polychronicity*. The average score should range from 4 to 5 (Bluedorn et al., 1999). The participants showed an average score of 3.19 (range [1.6, 5.1], *M*_Bayes_ = 3.20, CI [2.88, 3.52], *IPV* ~ Gaussian). Thus, their average value was below the expected average (Bluedorn et al., 1999).

### Self-monitoring

The ability of *self-monitoring in social situations* was assessed using a modified version of the *Self-Monitoring* Scale (Collani & Stürmer, 2009; originally by Snyder, 1974). The scale operationalizes four facets: *acting*, *other directedness*, *sensitivity to the reactions of others*, and *extraversion*, whereby larger values indicate a higher probability for *acting*, *other directedness*, *sensitivity to the reaction of others*, and *extraversion* in social contexts. On average, individuals should display percentage scores of 0.35, 0.46, 0.71, and 0.60 for *acting*, *other directedness*, *sensitivity to reactions of others*, and *extraversion*, respectively (Collani & Stürmer, 2009). To the best of our knowledge, this scale has not been used to directly investigate social traits in relation to multitasking, but it illustrated a suitable assessment tool in our investigation given that its items did not require a translation, it is freely accessible, and it operationalizes cognitive constructs potentially affecting multitasking (Mathieu et al., 2000). The participants displayed an average *acting* value of 0.51 (range [0.09, 0.82], *M*_Bayes_ = 0.54, CI [0.47, 0.6], *acting* ~ skewed normal), an average *other directedness* value of 0.50 (range [0.11, 0.89], *M*_Bayes_ = 0.49, CI [0.42, 0.56], *other directedness* ~ Gaussian), an average *sensitivity to the reactions of others* value of 0.41 (range [0, 1], *M*_Bayes_ = 0.4, CI [0.33, 0.48], *sensitivity to the reactions of others* ~ skewed normal), and an average *extraversion* value of 0.35 (range [0, 0.86], *M*_Bayes_ = 0.36, CI [0.29, 0.43], *extraversion* ~ Gaussian). Thus, their average *acting* (0.51 > 0.35) and *other directedness* scale (0.50 > 0.46) scores were larger, and their *sensitivity to the reactions of others* (0.41 < 0.71) and *extraversion* scores (0.35 < 0.60) were lower than expected (Collani & Stürmer, 2009).

## Laboratory assessment

### Media multitasking

During the laboratory assessment, information on *media multitasking* was assessed and the *media multitasking index* (MMI) computed thereof (Ophir et al., 2009). For this, participants filled in two surveys: one assessing information on the total amount of media usage, and another assessing the frequency with which different media had been used in conjunction with another media. For the latter, a grid is used whereby the frequency of each media multitasking pairing is indicated by means of a 4-point Likert response scale. Implementation of such a grid is difficult in *SoSci* survey, which is why we made an in-house *Python* application for this. Larger *MMI* values suggest that several different media had been frequently used in conjunction. The average *MMI* should fall in a range from 4 to 5 (Ophir et al., 2009). Individuals of this sample displayed an average *MMI* value of 2.85 (range [0.79, 7.7], *M*_Bayes_ = 2.84, CI [2.42, 3.25], *MMI* ~ exponentially modified Gaussian). Thus, their mean *MMI* value was below average (2.85 < 4) (Ophir et al., 2009).

### Math task

Subsequently, a math task was performed. Individuals were to solve 32 math problems by performing standard math operations, e.g., “1 + 1,” “2—7,” “3 × 3,” or “12:6.” These math problems were randomly chosen from a set of combinations where integers ranging from 1 to 12 were combined with a math operation such that the solution equalled a positive integer (including zero). Responses were given manually by pressing digit keys on a keyboard. Math equations were presented in the center of the screen for 8 s. For each correctly solved math equation, one point was rewarded. Thus, between 0 and 32 points could be acquired.

### Radio task

Afterward, a radio task was performed. For this, individuals had to memorize 32 radio signals consisting of 5 elements. These comprised a *unit* identifier, a *unit attribute*, a *direction*, *distance information*, and an *action description*, respectively. For instance, “Charlie,” “Friendly Unit,” “Eastward relative to your Position,” “600 m,” “no Movement”). Then, the radio messages were to be noted down by typing the elements using a keyboard. Participants had 22 s for this. Half of the messages were spoken by a female and half of them by a male voice, respectively. A point was rewarded for each correctly reproduced element. Thus, individuals could acquire between 0 and 160 points.

### Working memory processing abilities

Visual *working memory* abilities were assessed using a high-load visual working memory task (Friedrich et al., 2023). Individuals were to memorize a set of four fruits/vegetables presented in a row from left to right in the center of the screen and mentally sort them according to either color or size, depending on subsequent visual cues. The participants had to indicate whether the suggested position of one element of the original set matched the so newly mentalized order of fruits/vegetables. The participants had up to 4 s to indicate if this was the case or not using either the left or right mouse key, respectively. The participants’ working memory performance was operationalized by means of reaction times and accuracy. On average, the participants responded in 1.76 s (range [1.11, 2.66], *M*_Bayes_ = 1.75, CI [1.63, 1.87], *reaction time* ~ Gaussian) and with 70.48% accuracy (range [18.75, 93.75], *M*_Bayes_ = 69.84, CI [65.60, 74.07], *accuracy* ~ Gaussian).

### Multi-attribute task battery (MATB)

*Laboratory multitasking* was assessed using the open-source version of the MATB (Cegarra et al., 2020). The MATB comprises four subtasks to simulate a flight scenario. *Tracking* requires individuals to steer a circle as close to the center of a hair-cross as possible using a joystick. S*ystem monitoring* requires individuals to monitor a set of barometers and buttons. If arrows deviate too strongly from the mid-line, *F1*, *F2*, *F3*, or *F4* must be pressed, respectively. Also, if one of two buttons changes color, either *F5* or *F6* must be pressed. *Communication* requires individuals to listen to radio signals. If their identifier precedes a signal, they are supposed to memorize the radio signal to adjust the radio frequencies of corresponding radios using arrow keys. R*esource management* requires individuals to monitor a tank system such that two tanks reach/maintain optimal filling levels by dis-/enabling pumps connecting six tanks with each other. The participants performed each task as a single-task and one time as a multi-task condition. Their performance was operationalized by means of the probability of misses (*system monitoring* and *communication*) and the deviation from optimal levels either as a function of the root-mean-squared error (*tracking*) or the mean absolute difference (*resource management*), respectively.

## Military assessment

**Fig. 1:**
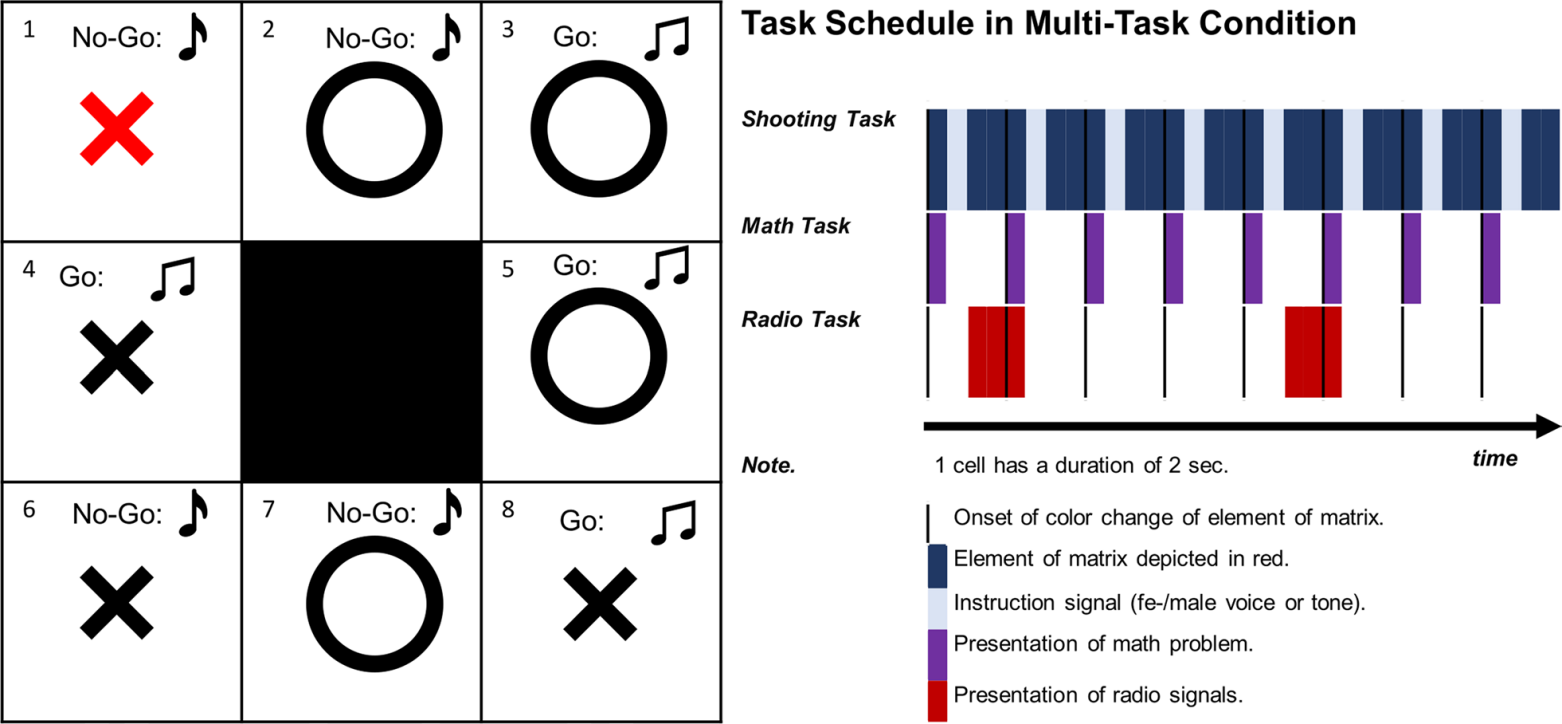
Shooting exercise. Left side: Individuals had to shoot at “O” targets and to ignore “X” non-targets. The color “red” indicated to which element individuals were supposed to attend to. In parallel to this, individuals knew that elements had to be attended to in a specific order starting from the upper left to the lower right cell. The numeration depicted here only serves illustrative purposes but was not displayed during the exercise. Elements were to be fought only if confirmed by an auditory signal: This could either be a (fe-)male voice telling “Go” or a high-pitched tone (as indicated by two notes for illustrative purposes). Otherwise, individuals were to refrain from shooting as indicated by a (fe-)male voice instructing to “No-Go” or a low-pitch tone (as indicated by one note for illustrative purposes). Thus, there were 2 true targets (cell 3 and 5), 2 true non-targets (cell 1 and 6), 2 false targets (cell 2 and 7), and 2 false non-targets (cell 4 and 8) in each 3 × 3 matrix. Right side: The shooting exercise was performed in a single- and multi-task condition. In the multitasking condition, individuals had to solve 8 math problems and memorize two radio signals consisting of 5 elements in addition to performing the shooting task

*Military multitasking* was assessed by combining a shooting exercise with the math and radio tasks described above. The shooting exercise required individuals to shoot/refrain from shooting at cells of a 3 × 3 matrix, wherein 4 cells represented targets and 4 non-targets. The central cell of the matrix represented a neutral element and had to be ignored at all times. Whether cells represented targets or non-targets was indicated by a visual display of the matrix on the screen of a Windows tablet, whereby an “O” indicated a target and an “X” a non-target, respectively. Participants lay on a ledge and shot with a modified Heckler & Koch G36 firearm air-compressed projectiles. The tablet was mounted to their left side. Cells had to be attended from the upper left to the lower right. The to-be-attended cell was indicated by a color change from white to red. Importantly, individuals were to wait for confirmation to fight any cell. This was administered by an auditory signal via headphones shortly after a cell element changed color. If the element indeed had to be fought, either a female or male voice said “Go” or a high-pitched tone was presented; otherwise, “No-Go” was said or a low-pitch tone played. Thus, 2 elements represented true targets, 2 true non-targets, 2 false targets, and 2 false non-targets. Participants could gain 1 point for each correctly fought/ignored element, resulting in between 0 and 8 points per matrix. See Fig. 1 for an illustration.

The shooting exercise was performed either as a single- or multi-task. In the multi-task condition, the participants had to solve math equations and memorize radio signals in addition to the shooting exercise. Thus, every time participants were supposed to shoot or refrain from shooting, a math equation was presented next to the matrix on the Windows tablet for 2 s. Subsequently, the auditory instruction to either fight or ignore the element was provided. Following this, radio signals were presented via headphones. The radio transmission was completed by approximately 6 s. After 8 s, individuals were to attend to the next cell and another math equation was presented. Thus, the participants had 8 s to shoot and solve the math equation. The next radio transmission was presented after 26 s. Responses to secondary tasks were provided verbally and noted down by an assistant. In total, individuals were supposed to solve 8 math equations and to memorize two radio signals (of 5 elements) per matrix in the multi-task condition. See Fig. 1 for an illustration (right side).

In the single-task condition, three matrices had to be fought whereby the instruction to shoot/refrain from shooting was indicated by either a male or female voice or a tone signal, respectively. Then, the participants performed the shooting task as a multi-task condition in 8 blocks. One block consisted of two matrices. Blocks differed depending on whether the radio signal was spoken by a male/female voice or a tone was displayed. In total, the participants performed 2 blocks where a male or female voice presented the radio signal and four blocks where a tone was displayed, respectively. Participants were asked to take a break between blocks. For this, they got up and walked around an administration station. This procedure also prevented that the participants’ shooting performance was confounded by keeping the firearm in a fixed position. The sequence according to which stimuli had been presented was random; and different stimulus materials were used in each assessment condition.

## Statistical procedures and data availability

### Hypothesis testing

We hypothesized that (1) *laboratory multitasking* performance predicts *military multitasking*; and (2) that this relationship was mediated by *working memory processing abilities*. *Laboratory multitasking* was operationalized by means of MATB task performance. We computed a *MATB compound score* exclusively based on the performance in the multi-task condition; additionally, we computed a *multitasking throughput* (MT) measure where standardized difference scores between single- and multi-task performance were averaged over each MATB task (see, Hilla et al., 2024, for further details). Moreover, we computed *MT*_Military_ values based on the shooting, math, and radio performance (as a function of probability of misses/errors). Hereby, negative and positive values indicate a decrement and improvement, respectively. *Working memory processing abilities* were operationalized based on reaction times (of correct trials) and percentage correct. To test our hypotheses, we conducted hierarchical Bayesian regression analyses and tested whether predictors explained a significant portion of the dependent variable using Bayes factors (*BF*_10_) by comparing the fit between data and models with predictors and without predictors. See Table 1 for an overview of the tested models. Only models with *BF*_10_ > 3 were further analyzed. There were three major advantages to this approach compared to classical statistical methods: firstly, most classical statistical methods rely on the assumption of normally distributed data/residuals to estimate effects/parameter values. Deviations of the data from this assumption may cause unreliable outcomes that are difficult to replicate. Bayesian procedures on the other hand can be based on many link functions aside from a Gaussian distribution allowing for modeling effects/parameter values of even extremely skewed data (by using e.g., exponentially modified Gaussian or skewed normal link functions). Given that many demographic and performance variables were skewed in our investigation, a Bayesian approach was best suitable for estimating parameter values thereof. Secondly, Bayesian procedures employ a more powerful re-sampling (of posterior distributions) than bootstrapping by considering priors and link functions; while bootstrapping distributions are based on random draws of the observed data. We used 4 Markov chains with at least 5000 iterations to generate Bayesian samples, which is sufficient to estimate (effect) parameter values appropriately. Thirdly, Bayes factors allow to quantify the likelihood of model fits and thereby of effects. For example, *BF*_10_ > 3 indicates that the effect is at least three times more likely than no effect. This is more informative than *p*-values and therefore preferable. A common concern when using Bayesian procedures though is the selection of proper priors and link functions. We chose priors based on a priori knowledge (e.g., public data on students’ age distributions at German universities) and data simulations in accordance with the observed data (e.g., we selected priors of parameter estimates such that a simple data simulation thereof would cover the range of a variable distribution of interest). We selected link functions (Gaussian, exponentially modified Gaussian, or skewed normal distributions) based on initial model fit evaluations (see, Hilla et al., 2024, for details). Bayesian parameter estimates are indicated by the subscript ‘Bayes’, e.g., *M*_Bayes_. In contrast, *M* and *SD* illustrate the mean and standard deviation determined by means of maximum likelihood procedure (see, Hilla et al., 2024, for details). All regression coefficients, *B*, were estimated using Bayesian procedures. Therefore, the subscript ‘Bayes’ was neglected. Moreover, the Abbreviation CI stands for credibility interval, which is the Bayesian equivalent to the confidence interval—but in contrast to the confidence interval, credibility intervals actually indicate the 95% most probable values of the posterior distribution. We controlled for the fit between Bayesian and observe data applying posterior distribution checks.

**Table 1:** Schematic model specifications

Procedure	Model	
Mediation	${\text{Multitasking}}_{{{\text{Military}}}} = \beta _{{{\text{direct}}}} \times {\text{Multitasking}}_{{{\text{Laboratory}}}} +\beta _{{\text{b}}} \times {\text{Working}} {\text{Memory}}$,	(1.1)
	${\text{Working Memory = }}\beta _{{\text{a}}} \times {\text{Multitasking}}_{{{\text{Laboratory}}}}$,	(1.2)
	$\beta _{{{\text{indirect}}}} =\beta _{{\text{a}}} \times\beta _{{\text{b}}}$,	(1.3)
	$$\beta _{{{\text{total}}}} =\beta _{{{\text{direct}}}} + \left( {\beta _{{\text{a}}} \times\beta _{{\text{b}}} } \right)$$	(1.4)
Control	${\text{Multitasking}}_{{{\text{Military}}}} { = }\beta _{{{\text{direct}}}} \times {\text{Multitasking}}_{{{\text{Laboratory}}}} { + }\beta _{{\text{b}}} \times {\text{Covariable}}$,	(2.1)
	${\text{Covariable = }}\beta _{{\text{a}}} \times {\text{Multitasking}}_{{{\text{Laboratory}}}}$,	(2.2)
Model Comparison	Null-Modell without random intercept: $Criterion = \beta_{0} + \varepsilon_{i,j}$, *i* = participant, *j* = condition	(3.1)
	Null-Modell with random intercept: $Criterion = \beta_{0} + \gamma_{i,j} + \varepsilon_{i,j}$, *i* = participant, *j* = condition	(3.2)
	Predictor-Modell with random intercept: $Criterion = \beta_{0} + \beta_{j} + \gamma_{i,j} + \varepsilon_{i,j}$, *i* = participant, *j* = condition	(3.3)

Multitasking_Military_: {Multitasking throughput (MT) based on single- and multi-task performance of the math, radio, and shooting tasks}; Multitasking_Laboratory_: {Multi-Attribute Task Battery (MATB) compound score (based on multi-task performance only), MT based on difference between MATB single- and multi-task performance}; Working Memory: {reaction time in seconds, percentage correct}; Covariables: {Age, Video Gaming Experience, Autism Spectrum Quotient, Perspective Taking, Empathic Concern, Extraversion (Big-5), Agreeableness, Conscientiousness, Neuroticism, Openness to Experiences, Inventory of Polychronic Values, Acting, Other Directedness, Sensitivity to the Reactions of Others, Extraversion (Self-Monitoring), Media Multitasking, Military Service Duration}

### Control analyses

Additionally, we conducted a series of control analyses: for instance, we controlled if there were indeed multitasking decrements in the MATB, math, radio, and shooting tasks and if these differences altered depending on the instruction condition (male vs. female vs. tone), respectively. For this, performance measures were z-standardized relative to the Bayesian mean estimate. Then, we tested to which model the data fit best (null model with random intercepts on the subject level only vs. model with condition (single- vs. multi-task) as predictor) using Bayes factors. Moreover, we controlled if the participants’ personality traits (operationalized based on BFI-K *extraversion*, *agreeableness*, *conscientiousness*, *neuroticism*, and *openness to experiences*; and *IPV polychronicity* scores), social traits (based on *AQ*; *perspective taking*, *empathic concern*, and Self-Monitoring Scale *acting*, *other directedness*, *sensitivity to the reactions of others*, and *extraversion* scores), demographic characteristics (*age* in years), media habits (*video gaming experience* as a function of average play time, *media multitasking* based on MMI), and /or military experience (*military service duration*) correlated with any of the multitasking and/or working memory performance measures; and thereby affected the outcome of our primary analyses (Blacker et al., 2019; Hilla et al., 2024; Himi et al., 2023; Matthieu et al., 2000; Schwarze et al., 2024). See Table 2 for an overview of these models. We did not control for gender-based differences given that we only had data sets of four individuals identifying as female (see Himi et al., 2023 for a discussion on gender-based differences in multitasking performance). Furthermore, we could not control for performance differences depending on military specialty/branch. This is unfortunate as we used multitasking performance measures directly relating to tasks performed by soldiers of different military branches, e.g., the MATB simulates a low-fidelity flight simulation and therefore may be easier for soldiers of the Air Force. However, only 10 participants of the sample were part of the Air Force. Thus, this subsample was too small to conduct inferential statistical procedures. Moreover, it would not quite make sense to descriptively compare performance measures between participants of different military branches as their subsample size was too small and unbalanced.

### Missing data

Missing values due to technical issues were substituted by Bayesian mean estimates (i.e., 1 radio multi-task performance value, 3 MATB multi-task performance values, and 1 system monitoring performance value).

### Software and data availability

All statistical analyses were conducted by means of *R* statistical software in *R Studio* (R Core Team, 2024). Bayesian procedures were conducted using the *brms* package (Bürkner, 2018). Graphical visualizations were made using the *ggplot2* and *RColorBrewer* packages (Neuwirth, 2022; Wickham, 2016). The data supporting the findings of this study are available on request from the corresponding author. The data are not publicly available as they contain information that could compromise the privacy of research participants. The code supporting our analyses, detailed information on the priors, and experimental materials will be available on Open Science Framework (OSF) (https://osf.io/pmyec).

## Results

None of the models indicating a mediating effect of *working memory* on the association between *laboratory multitasking* and *military multitasking* were sufficiently supported by the data (*BF*_10_ < 3). In fact, neither *MT*_MATB_ nor *MATB compound* values correlated positively with *MT*_Military_ values, nor did any working memory performance measure correlate sufficiently large with any multitasking measure (see Table 2 for estimates).

**Table 2:** Correlations between multitasking and working memory measures

	Military Multitasking	Laboratory Multitasking		Working Memory	
	*MT*_Military_	*MT*_*MATB*_	*Compound Score*	*%*	*RT*
*MT*_Military_	1	− 0.12	− 0.2	− 0.15	0.04
*MT*_MATB_		1	0.77	− 0.18	− 0.07
*Compound Score*			1	− 0.06	− 0.15
*%*				1	− 0.13
*RT*					1

Values indicate Pearsons’s correlation estimates *r*. *MT*: Multitasking Throughput; *MATB*: Multi-Attribute Task Battery, *%*: Percentage Correct; *RT*: Reaction Time in s

**Fig. 2:**
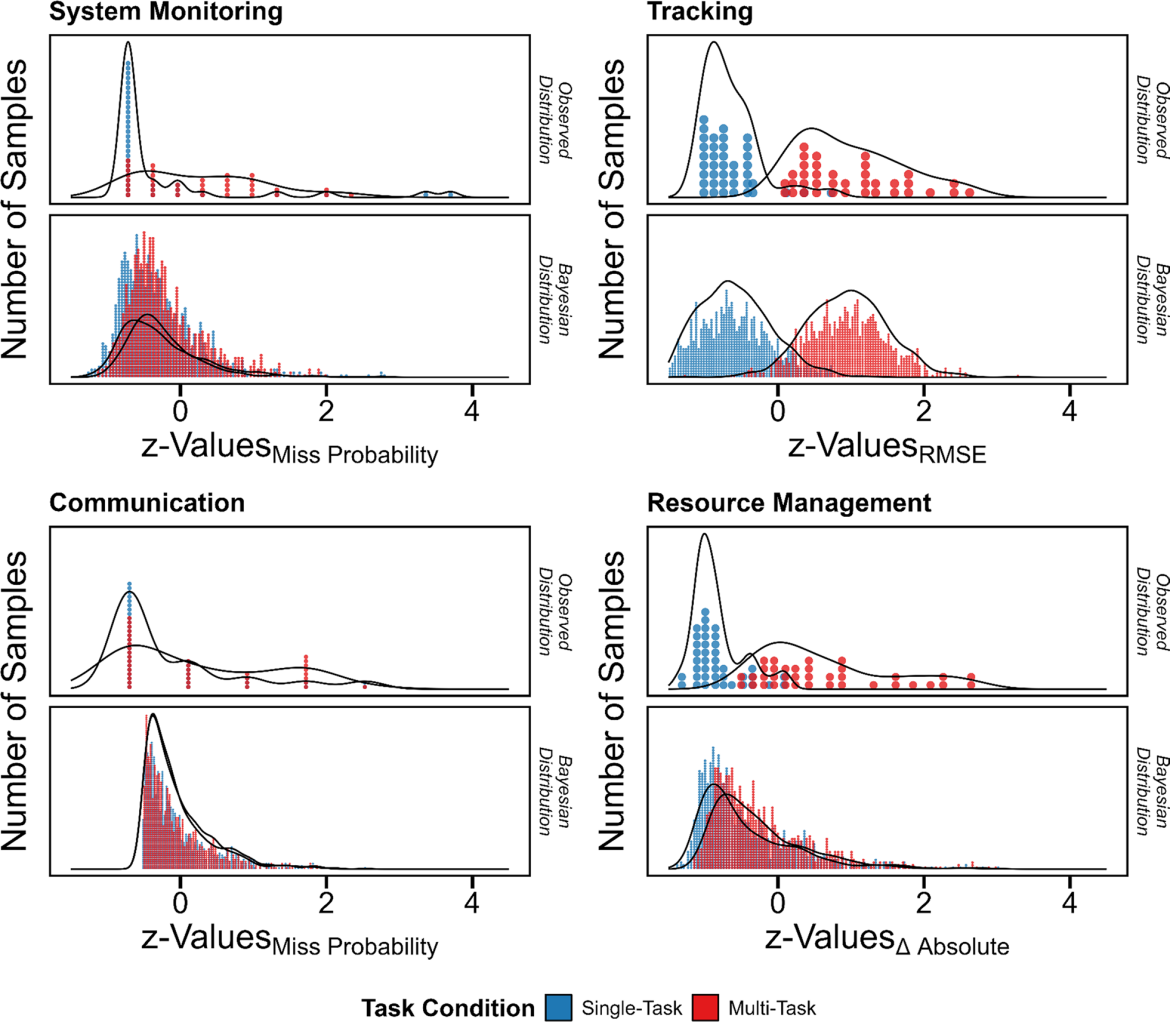
Differences in multi-attribute task battery performance measures between single- and multi-task conditions. System Monitoring and Communication task performance were operationalized based on the probability of misses, and Tracking and Resource Management task performance based on the root-mean-squared error (RMSE) and absolute difference scores, respectively. Performance measures were z-standardized with respect to the corresponding Bayesian mean estimate. Exponentially modified Gaussian distributions served as link functions. Blue and red hue indicate single- and multi-task performance, respectively. Larger values indicate worse performance. Performance was worse in the multi- vs. single-task condition in all tasks except the Communication task

Nevertheless, MATB task performance differed between task conditions (*system monitoring*: *BF*_10_ = 7.30, *tracking*: *BF*_10_ > 100; *communication*: *BF*_10_ = 0.14; *resource management*: *BF*_10_ > 100): Performance was worse in the multi- than the single-task condition in the *system monitoring*, *tracking*, and *resource management* tasks, and there was no difference in the *communication* task, respectively (see Table 3 and Fig. 2).

**Table 3:** Differences in multi-attribute task battery performance measures between single- and multi-task conditions

Task	Parameter Label	*B*	*SE*	95%-*LB*	95%-*UB*
System Monitoring	Intercept	− 0.32	0.05	− 0.41	− 0.23
	Multi-Task	0.15	0.06	0.02	0.27
	Sigma	0.19	0.01	0.17	0.21
	Beta	0.51	0.01	0.49	0.53
Tracking	Intercept	− 0.64	0.08	− 0.79	− 0.48
	Multi-Task	1.59	0.12	1.35	1.83
	Sigma	0.50	0.01	0.48	0.52
	Beta	0.20	0.01	0.18	0.22
Communication	Intercept	− 0.05	0.01	− 0.08	− 0.03
	Multi-Task	0.01	0.01	− 0.01	0.03
	Sigma	0.01	0.00	0.01	0.01
	Beta	0.46	0.01	0.44	0.48
Resource Management	Intercept	− 0.42	0.03	− 0.48	− 0.35
	Multi-Task	0.17	0.05	0.08	0.27
	Sigma	0.10	0.01	0.09	0.12
	Beta	0.71	0.01	0.69	0.73

Estimates were based on exponentially modified Gaussian link functions. *SE*: standard error; 95%-*LB*: lower bound of 95%-credibility interval; 95%-*UB*: upper bound of 95%-credibility interval. All values were z-standardized. Positive *B* values indicate a decrement and negative values an improvement. Sigma and Beta illustrate distribution parameter estimates of the exponentially modified Gaussian distribution

**Fig. 3:**
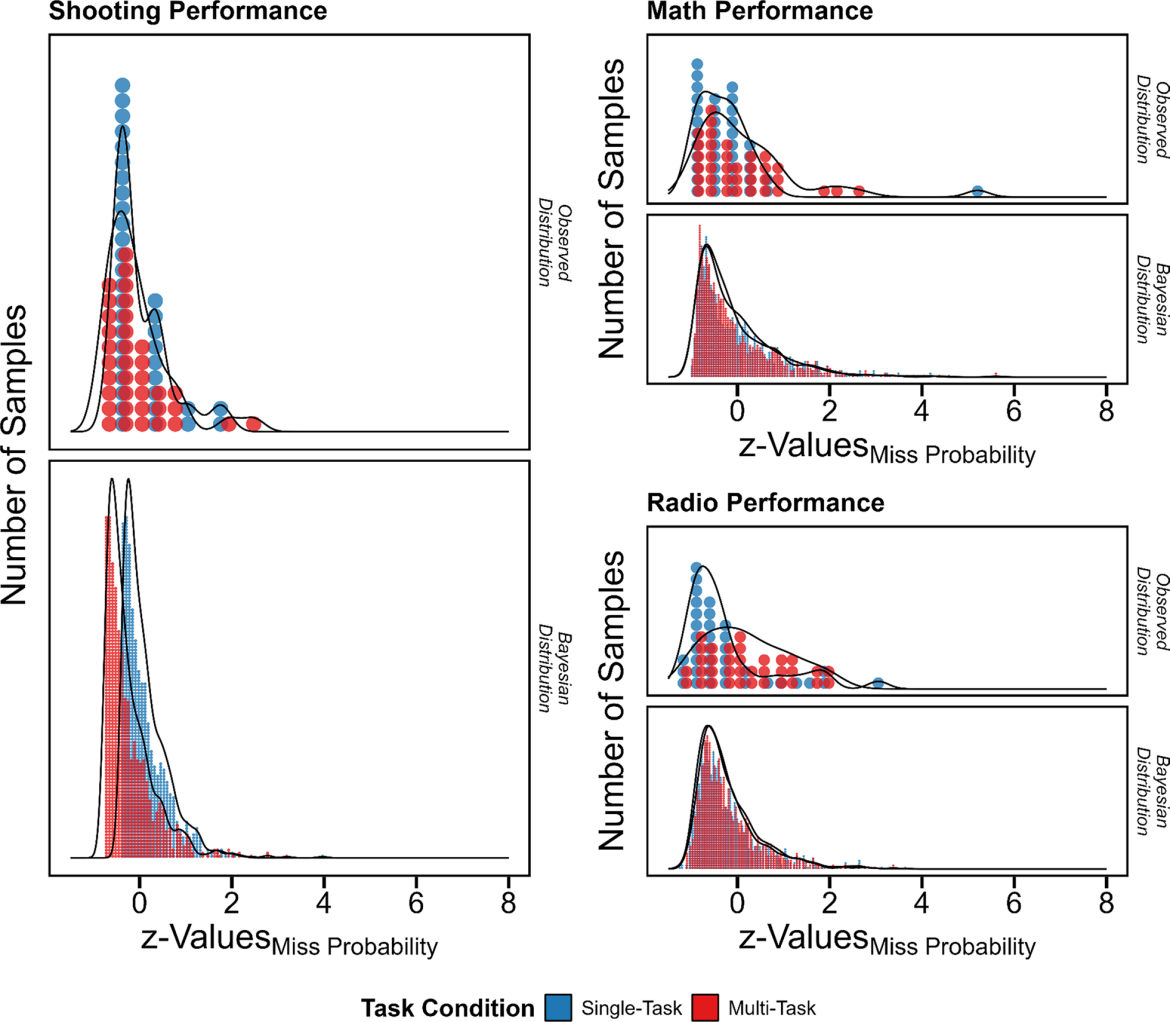
Differences in military task performance between single- and multi-task conditions. Probability of misses (errors) was used as performance measure of the shooting, math and radio tasks. Performance measures were z-standardized with respect to the corresponding Bayesian mean estimate. Exponentially modified Gaussian distributions served as link functions. Blue and red hue indicate single- and multi-task performance, respectively. Larger values indicate worse and smaller good performance. In the shooting task, performance was better in the multi- than single-task condition. In the math task, performance was similar irrespective of the condition. In the radio task, performance was slightly worse in the multi- than single-task condition

Furthermore, *shooting* performance differed between conditions (*BF*_10_ > 100 cf. null model; *BF*_10_ = 66.60 cf. second-best model). The participants performed better in the multi- than single-task condition—an effect probably related to practice given that the average error decreased during time on task (*r* = -0.69). *Math* performance was similar across all conditions; and there was a slight indication of a performance decrement in the multi- compared to the single-task condition in the *radio* task (see Table 4 and Fig. 3).

**Table 4:** Military operation performance differences in shooting, math, and radio task performance in single- vs. multi-task conditions

Tasks	Parameter Label	*B*	*SE*	95%-*LB*	95%-*UB*
Shooting	Intercept	0.12	0.02	0.08	0.14
	Multi-Task Condition	− 0.36	0.01	− 0.38	− 0.33
	Sigma	0.00	0.00	0.00	0.01
	Beta	0.50	0.01	0.48	0.52
Math Solving	Intercept	− 0.06	0.04	− 0.13	0.02
	Multi-Task Condition	0.00	0.01	− 0.02	0.02
	Sigma	0.05	0.00	0.04	0.06
	Beta	0.85	0.04	0.77	0.93
Radio Memorizing	Intercept	− 0.24	0.06	− 0.35	− 0.12
	Multi-Task Condition	0.06	0.04	− 0.03	0.14
	Sigma	0.15	0.05	0.05	0.24
	Beta	0.65	0.04	0.57	0.73

Estimates were based on exponentially modified Gaussian link functions. *SE*: standard error; 95%-*LB*: lower bound of 95%-credibility interval; 95%-*UB*: upper bound of 95%-credibility interval. All values were z-standardized. Positive *B* values indicate a decrement and negative values an improvement. Sigma and Beta illustrate distribution parameter estimates of the exponentially modified Gaussian distribution

**Fig. 4:**
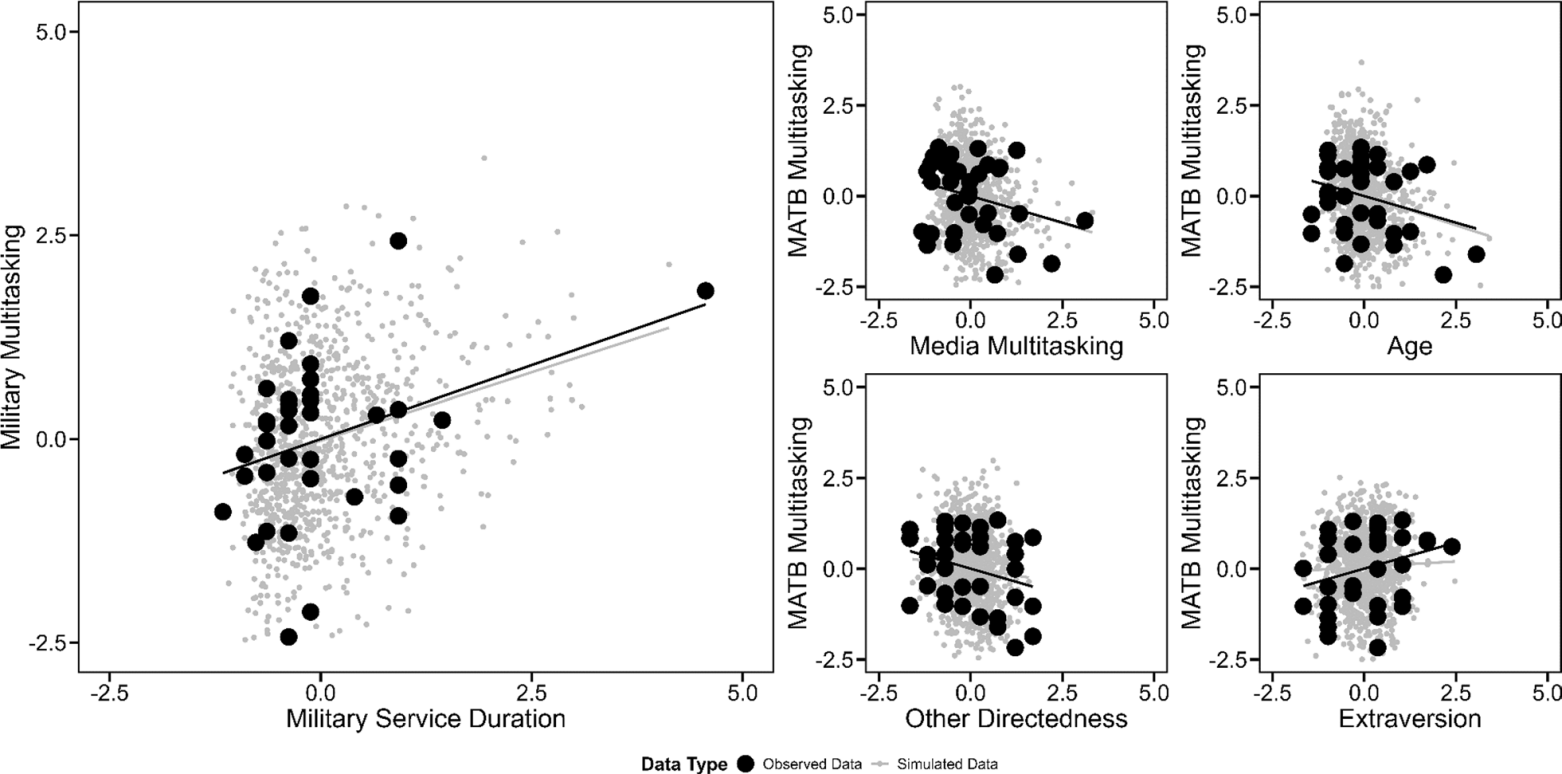
Associations between multitasking measures and control variables. Left side: Association between military multitasking and military service duration. Military multitasking was operationalized based on a multitasking throughput measure accounting for performance differences between single- and multi-task conditions in a shooting, math, and radio task. Left side: Association between Multi-Attribute Task Battery (MATB) performance and media multitasking (upper left panel), age (upper right panel), other directedness (lower left panel), and extraversion (lower right panel). MATB performance was derived from a compound score encompassing four subtasks when all tasks had to be performed simultaneously. Increasing slopes indicate a positive effect. All values were z-standardized. Black data points indicate the participants’ actual data. Gray data points were simulated based on Bayesian estimates

*Military service duration* explained a moderately large portion of the variance of *military multitasking*, *r* = 0.36, *BF*_10_ = 8.71. Thus, individuals were better at *military multitasking* the more years they had already served in the military (see Fig. 4 (left side) for a visualization). On top of that, *MATB compound scores* were moderately explained by *media multitasking*, *r* = -0.29, *BF*_10_ = 3.83, *age*, *r* = -0.29, *BF*_10_ = 3.85, and *Self-Monitoring Scale other directedness*, *r* = -0.29, *BF*_*10*_ = 3.50, and *extraversion* scale scores, *r* = 0.28, *BF*_*10*_ = 3.46, respectively. Thus, *MATB* performance (in the multi-task condition) was worse in individuals the more habitually they used several media simultaneously (*media multitasking*), the older they were (*age*), and the more they relied on input of others (*Self-Monitoring Scale other directedness*); but also, better in individuals with high extraversion in social contexts (*Self-Monitoring Scale extraversion*). See Fig. 4 (right side) for a visualization of these correlations. No additional correlations between other co-variates and multitasking and/or working memory measures were significant. Also, there were no meaningful differences in military multitasking performance depending on whether a (fe-)male voice or a tone signal had been presented.

## Discussion

We hypothesized that (1) *laboratory multitasking* would predict *military multitasking*, and that (2) this association would be mediated by *working memory processing abilities*. In contrast, we found weak and negative associations between *military* and *laboratory multitasking*, and multitasking and *working memory* measures, respectively. Aside this, we observed correlations between *military multitasking* and *military service duration*, and between *laboratory multitasking* and *media multitasking, age*, and *Self-Monitoring Scale other directedness* and *extraversion*, respectively.

## Military experience predicts military performance

Thus, we could not replicate the previous observation according to which flight simulation (MATB) task performance predicted military multitasking (Hilla et al., 2024). This original effect had been found in a relatively small sample of 25 individuals and was therefore at risk of representing a chance result. Our study outcome (being based on a larger sample) appears to confirm this suspicion. Likewise, differences in experimental designs between both investigations might constitute an alternative explanation. For instance, in this study, the participants performed a (“shoot/don’t shoot”) shooting exercise simulating conditions as experienced by sharpshooters. In contrast, in Hilla et al.'s (2024) study, individuals shot at ring targets. Thus, the participants relied on slightly different task representations when it came to the shooting task. It is important to note this because—as discussed above—multitasking performance is constrained by task representations (Musslick & Cohen, 2021): thus, performance decrements in multitasking conditions are typically related to interferences between at least two tasks due to shared resources. One approach to overcome this issue is practice (Garner & Dux, 2023; Musslick & Cohen, 2021). To illustrate this, many drivers might remember that learning to drive a car was exhausting until one day ‘it suddenly just happened automatically’. An explanation for this may be that interference between task representations in a multitasking condition is resolved by acquiring a new task representation unifying the original ones (Garner & Dux, 2015). In this regard, we speculate that the participants in this study (being officer cadets) might have already acquired a task representation suitable for performing our simulated military operation. This is because, for acquiring the officer rank, individuals must pass at least basic military training of one year. The correlation between military multitasking and military service duration supports this argument. In contrast, in the study by Hilla et al. (2024), there might have been a lack of such a task representation given that the experimental design was more artificial; therefore, there may have been more task interference which may be predictable by means of MATB performance. To confirm this, further research should test this hypothesis in freshmen and in samples of different military branches.

## Multitasking performance depends on task constraints (not requirements)

Given that working memory has been shown to mediate the relationship between laboratory and military multitasking (Colom et al., 2010; Hambrick et al., 2011) and to predict multitasking even when attention control was controlled for (Redick et al., 2016), we hypothesized that working memory constitutes an essential cognitive substrate of (military) multitasking behavior. However, we could not find sufficient evidence in support of this hypothesis (see Biggs et al., 2015; Hamilton et al., 2019; Hilla et al., 2024 for similar results). This raises the question of whether working memory should be discarded as a cognitive substrate of (military) multitasking? We do not believe so. Rather, we propose a stronger distinction between task *requirements* and *constraints* when discussing the role of cognitive functions in (military) multitasking. For instance, it is not possible to execute either the MATB or a military operation without temporarily storing and updating information. Hereby, working memory constitutes a *requirement* of task performance, which does not necessarily bias how efficiently multitasking is being executed. In contrast, multitasking proficiency rather depends on how efficiently operations of cognitive functions are being executed. For instance, for executing the MATB, our simulated military operation, and the working memory task short radio signals, fairly long and complex radio signals, and naturalistic visual input had to be memorized and processed, respectively. This required employing either the same memory operations but with different effort or different operations. However, for MATB, military, and working memory task performance to correlate all three tasks would have needed to be biased by the same operations of working memory. Thus, working memory operations illustrate *constraints* of task performance. In conclusion, working memory illustrates a task *requirement* but not necessarily a *constrain* in multitasking conditions, which may explain inconsistencies regarding the role of working memory in the literature.

## Additional predictors of multitasking performance

On top of that, MATB performance was negatively correlated with *media multitasking* and *age*. *Media multitasking* has already been linked to an increased distractibility and impaired task-switching abilities (Ophir et al., 2009). In this regard, a negative relation with MATB performance makes sense, given that Stasch and Mack (2023) found that strategic (attention) shifting affected MATB performance. On top of that, reduced task-switching proficiency correlates with age (Schwarze et al., 2024). However, this result should not be taken too seriously as the participants in this study were young adults unlikely displaying any serious cognitive impairment due to age. It is interesting though that we found a negative correlation between MATB and MMI scores, given that the participants exhibited a notably smaller propensity for *media multitasking* than civilian individuals (Ophir et al., 2009). This could indicate that media multitasking may be particularly disadvantageous for military performance. Thus, media multitasking should be considered more prevalently in future research.

Besides, the participants displayed high AQ values. This may indicate impaired working memory processing abilities (Friedrich et al., 2023). For this reason, we performed an exploratory analysis with our data, which indicated (semantically speaking) negative relationships between AQ scores and working memory performance. However, these effects had been too small to constitute a plausible explanation for our results. Furthermore, AQ values did not significantly affect the individuals’ multitasking performance. Thus, AQ unlikely biased our results.

Aside this, we observed that the participants displayed noticeably smaller IPV (*polychronicity*) values (Bluedorn et al., 1999), and larger Self-Monitoring Scale *acting* and *other directedness* and smaller *sensitivity to the reactions of others* and *extraversion* scores than civilian individuals (Collani & Stürmer, 2009). Himi et al. (2023) suggested that *polychronicity* may positively affect multitasking. Thus, it may be possible that a small affinity for *polychronicity* may negatively impact on multitasking. However, our control analyses did not support such a concern. In contrast, we found that Self-Monitoring Scale *other directedness* and *extraversion* values negatively and positively correlated with MATB performance, respectively. This was interesting, given that the participants displayed larger *other directedness* and smaller *extraversion* values than civilians (Collani & Stürmer, 2009), which according to our observed correlations could be interpreted as social traits disadvantageous for multitasking performance. But this conclusion may be premature, given that there is a high chance that the participants’ reports may have been confounded by expectation effects. It was clear to all participants that the study aimed to investigate predictors of military performance. Thus, we speculate that the participants might have indicated exaggerated responses to the Self-Monitoring Scale items to comply with military values (e.g., loyalty, duty, etc.). Furthermore, these associations should be considered with caution as an exploratory analysis indicated that the data did not fit well to the proposed scales (Collani et al., 2009). Thus, these correlations have a high probability of constituting chance results.

Furthermore, we speculate that experimenter bias may have also contributed to the participants’ relatively larger empathy scale scores. However, given that neither their *empathic concern* nor *perspective taking* scale scores correlated with any of the performance measures, we do not consider them problematic.

## Implications

The observation that the participants’ military experience (as a function of *military service duration*) explained military performance best, constitutes the most important finding of this study. It indicates that military proficiency may be affected by a priori military training (even in officer cadets). At first glance, this appears to be trivial because one would certainly expect military training to impact on military performance. However, according to the literature review on training effects on military performance (Blacker et al., 2019) and the experimental work discussed previously (Biggs et al., 2015; Hamilton et al., 2019), training effects should predominantly apply to military conditions as a function of *near transfer* when there are similarities between the training and the task. In contrast, we observed that merely the *duration* of military service was indicative of military multitasking proficiency—an astonishing outcome, given that this effect was observed in a mixed sample of soldiers (of the Infantry, Air Force, Navy, Cyber Security, and Medical Service branches). Nevertheless, this association appears to be reliable, given that it had already been observed and hence replicated (Hilla et al., 2024).

Then again, Biggs et al.’s (2015) and Hamilton et al.’s (2019) and our shooting tasks were quite similar (being “shoot/don’t shoot” tasks). Following Blacker et al.’s (2019) logic, this implies two things: firstly, inhibitory control, quick decision-making, and processing speed may illustrate *constraints* of military performance (at least when they are characterized by “shoot/don’t” shoot requirements); and secondly, military experience based on military service duration may serve a similar function in our study as the cognitive training did in Biggs et al.’s (2015) and Hamilton et al.’s (2019) studies, which is improving military performance. However, these implications are based on conceptual considerations not quantitative data directly supporting this hypothesis. Nevertheless, our observations that participants displayed no multitasking decrements in the military task but practice related improvements indirectly supports the account as both effects combined suggest that the participants may have acquired a holistic task representation to perform the shooting task (Garner & Dux, 2023; Musslick & Cohen, 2021).

Despite that, we would not advocate that future research should assess/train inhibitory control, quick decision-making, and processing speed (in contrast to working memory) as predictors of military performance or mediators of associations between cognitive/multitasking measures and military performance. Instead, we propose that such measures should be used as co-variates to gain more insights into the association between military characteristics (e.g., military service duration, military branch, etc.) and military performance. This might come as a surprise, given that identifying laboratory measures for predicting military performance illustrated the main aim of this study. And indeed, we had reasonable cause for this, given that there is a need for laboratory predictors of military performance as field assessments are challenging or not applicable at all. In fact, some paradigms were developed and/or used for this purpose, e.g., the *SynWin* (Elsmore, 1994; Hambrick et al., 2010, 2011) and MATB (Cegarra et al., 2020; Hilla et al., 2024). But then again: why should we consider a laboratory measure as more important than a military variable when its standardization causes unwanted variance confounding the predictability of military performance? This is not to say, that we should ignore the fact that military service duration poses an insufficiently defined construct (for predicting military performance). But it appears to illustrate a predictor of military performance unconfounded by experimenter and laboratory influences.

As for a practical implication, our results suggest that rather the cadets’ training progress as opposed to their cognitive capabilities may be predictive of their military performance. Hence, personnel selection procedures might need to consider cognitive capabilities as less important and rely more on training achievements.

## Limitations and restricted generalizability

We found the aforementioned effects predominantly in officer cadets. There were also individuals with more military experience. However, we had no further details on them. Furthermore, the individuals of this sample were mostly affiliated to the Infantry. Thus, it is difficult to predict what aspects of military training contributed to the effects and if and how these translate to individuals with different military education. On top of that, we observed the effects in a sample consisting predominantly of individuals of male gender. Therefore, we cannot simply conclude that our effects translate to individuals of female gender (but see Himi et al., 2023 for a discussion).

Besides, we discussed that attention/inhibitory/cognitive control, executive functions, processing speed, and (quick) decision-making alongside working memory play a considerable role in (military) multitasking (Biggs et al., 2015; Hamilton et al., 2019; Himi et al., 2019; Musslick & Cohen, 2021; Stasch & Mack, 2023). Nevertheless, we only investigated the impact of working memory processing on (military) multitasking. Thus, we neglect a notably large portion of potential cognitive co-variates. We did this because a large and convincing body of research highlighted the significance of working memory in (military) multitasking (Colom et al., 2010; Hambrick et al., 2011; Redick et al., 2016). Assessing additional cognitive measures would not have been reasonable as participants in this study already participated in three assessments at different locations and at different time points. To maintain compliance and to prevent dropouts, we administrated as little additional assessments as possible. Moreover, the German privacy policy for data processing provides that only data required for a research aim (and in accordance with the research plan provided to the Ethics committee) may be acquired and processed. Therefore, we did not include additional cognitive assessments. But future research should address this shortcoming.

## Conclusion

In conclusion, it appears unlikely that one can simply predict military performance by means of laboratory measures. Such associations are likely restricted by task *constraints* (rather than *requirements*) and task representations as a function of practice and experience. Thus, military training progress and experience may illustrate promising predictors of military performance. In this regard, future research should investigate the role of cognitive functions as co-variates rather than as predictors of associations between military characteristics and performance to gain insights into how military task representations are acquired.

## Data Availability

The data supporting the findings of this study are available on request from the corresponding author, MS. The data are not publicly available as they contain information that could compromise the privacy of research participants. The code supporting our analyses and experimental materials will be available on Open Science Framework (https://osf.io/pmyec/).
